# Bone Structural Parameters in Adults with Cystic Fibrosis: Contribution of Adherence to the Mediterranean Diet

**DOI:** 10.3390/jcm15062366

**Published:** 2026-03-19

**Authors:** María Carmen Andreo-López, Victoria Contreras-Bolívar, María Dolores San Matías-Marín, José Alejandro Ávila-Cabreja, Alberto Suárez-Catalina, Francisco Casas-Maldonado, Silvia Merlos-Navarro, Casilda Olveira, Gabriel Olveira, José Manuel Vaquero-Barrios, Manuel García-Amores, Marta Bravo-Martínez del Valle, Diego Becerra-García, Manuel Muñoz-Torres

**Affiliations:** 1 Endocrinology and Nutrition Unit, University Hospital Clínico San Cecilio, 18016 Granada, Spain; mcandreo21@correo.ugr.es (M.C.A.-L.); mmt@ugr.es (M.M.-T.); 2 Instituto de Investigación Biosanitaria de Granada (Ibs.Granada), 18012 Granada, Spain; javila@fibao.es; 3 Institute of Health Carlos III, CIBER of Frailty and Healthy Aging (CIBERFES), 28029 Madrid, Spain; 4 Nuclear Medicine Unit, University Hospital Clínico San Cecilio, 18016 Granada, Spain; dolores.sanmatias.sspa@juntadeandalucia.es (M.D.S.M.-M.); diego.becerra.sspa@juntadeandalucia.es (D.B.-G.); 5 Clinical Analysis Unit, University Hospital Clínico San Cecilio, 18016 Granada, Spain; alberto.suarez.sspa@juntadeandalucia.es; 6 Respiratory Medicine Unit, University Hospital Clínico San Cecilio, 18016 Granada, Spain; francisco.casas.sspa@juntadeandalucia.es; 7 Respiratory Medicine Unit, University Hospital Virgen de las Nieves, 18014 Granada, Spain; silvia.merlos.sspa@juntadeandalucia.es; 8 Respiratory Medicine Unit, Hospital Regional of Málaga, 29010 Malaga, Spain; casi1547@separ.es; 9 Instituto de Investigación Biomédica de Málaga-Plataforma Bionand (IBIMA), 29590 Malaga, Spain; gabrielm.olveira.sspa@juntadeandalucia.es; 10 Department of Medicine, University of Málaga, 29071 Malaga, Spain; 11 Institute of Health Carlos III, CIBER of Diabetes and Associated Metabolic Diseases, 28029 Madrid, Spain; 12 Respiratory Medicine Unit, University Hospital Reina Sofía, 14004 Cordoba, Spain; josem.vaquero.sspa@juntadeandalucia.es; 13 Respiratory Medicine Unit, University Hospital of Jaén, 23007 Jaen, Spain; manuel.garcia.amores.sspa@juntadeandalucia.es; 14 Radiology Unit, Hospital Regional of Málaga, 29071 Malaga, Spain; marta.bravo.sspa@juntadeandalucia.es; 15 Department of Medicine, University of Granada, 18016 Granada, Spain

**Keywords:** cystic fibrosis, bone mineral density, 3D-DXA, trabecular bone score and mediterranean diet

## Abstract

**Background:** Cystic fibrosis-related bone disease (CFBD) is a comorbidity whose prevalence is increasing. Conventional areal DXA (aDXA) only assesses part of bone strength, whereas new techniques provide a more comprehensive assessment of bone structure. However, these tools are rarely implemented systematically. Furthermore, the contribution of clinical factors to bone structural parameters remains insufficiently characterized. Therefore, this study aimed to comprehensively assess cortical and trabecular bone alterations in adults with CF, and to explore clinical and lifestyle factors associated with bone health. **Methods:** A case–control study was conducted including 32 adults with CF and 70 healthy controls matched by age, sex, and BMI. Bone status was assessed using aDXA, trabecular bone score (TBS), and three-dimensional DXA (3D-DXA). Clinical data, body composition, lifestyle variables, and biochemical markers were collected. Multivariate linear regression models were applied to explore factors associated with bone parameters. **Results:** Twenty-two percent of CF people had prevalent fragility fractures and the percentage with low bone mass was significantly higher (28.1% vs. 5.7%; *p* < 0.001). TBS categories indicated more degraded microarchitecture in CF (*p* = 0.002). Cortical and trabecular 3D-DXA values were also significantly lower (*p* < 0.001). Adherence to the Mediterranean diet (MedDiet) was significantly associated with most of the bone parameters evaluated. Other variables associated with bone parameters included sex, fat-free and fat mass, prolonged corticosteroid use, intake of oral nutritional supplements, FEV_1_, disease duration and P1NP-values. **Conclusions:** Adults with CF revealed significant cortical and trabecular structural deficits. Among the evaluated factors, adherence to MedDiet was associated with several bone parameters, suggesting a potential role of dietary patterns in CF bone health. Further studies are needed to confirm these preliminary results.

## 1. Introduction

Advances in CF clinical care and treatment have markedly improved survival, raising life expectancy from 10 years in 1962 to 68 years for individuals born in 2024 [1]. As people with CF live longer, medical complications such as cystic fibrosis–related bone disease (CFBD) can substantially increase their morbidity and mortality [2].

CFBD is manifested as compromised bone mineral density (BMD), bone quality, or both, leading to increased bone fragility [2]. The prevalence of CFBD rises with age. According to the 2024 CFF (Cystic Fibrosis Foundation) Patient Registry, 8.8% of adults aged >18 years with CF had a diagnosis of osteoporosis, increasing to 30% among those aged >60 years [1]. Furthermore, fracture risk in people with CF is higher than in the general population, with fracture rates ranging from 20% to 60% [1,3]. Fractures most commonly affect the ribs and vertebrae and may impair pulmonary function [2].

CFBD is diagnosed on the basis of fracture occurrence or low BMD on dual-energy X-ray absorptiometry (DXA) [2]. DXA is the gold standard for assessing areal bone mineral density (aBMD). However, aBMD reflects only part of bone strength, as it is a two-dimensional technique. Likewise, DXA cannot distinguish between trabecular and cortical bone, nor can it evaluate bone quality or microarchitecture—key components of bone strength—which limits the accuracy of fracture risk estimation [4]. Nevertheless, new techniques such as peripheral quantitative computed tomography (pQCT) have emerged, providing volumetric (three-dimensional) measurements of BMD (vBMD) and differentiating between bone compartments—cortical and trabecular. In fact, pQCT in adults with CF has improved the understanding of bone quality and strength [4]. The combination of pQCT and DXA can be highly sensitive for estimating fracture risk [3]. However, pQCT remains mainly a research tool due to its high cost and limited availability [4].

As an alternative, vBMD captured by three-dimensional DXA (3D-DXA) has shown excellent correlation with that measured by pQCT [5]. 3D-DXA is a novel technique that reconstructs femoral geometry and compartment-specific density (cortical bone and trabecular macrostructure) from standard hip DXA scans [5]. However, no studies have yet evaluated CFBD using 3D-DXA. In addition, bone microarchitecture estimation at the lumbar spine (LS) can be assessed from conventional DXA scans by Trabecular Bone Score (TBS) [4]. The information available on TBS in people with CFBD is very limited [6].

Identifying the factors associated with bone structural parameters (aBMD, TBS, vBMD) may help mitigate the consequences of CFBD. Potential risk factors include poor pulmonary function, chronic systemic inflammation, prolonged glucocorticoid use, malnutrition, the ΔF508 genotype, and reduced muscle mass and physical activity [7,8,9,10,11,12].

To date, no study has comprehensively assessed bone status in adults with CF using a combination of 3D-DXA, TBS, and conventional DXA. The present study aimed to characterize cortical and trabecular bone deficits in CF and explore clinical and lifestyle factors associated with bone health.

## 2. Materials and Methods

### 2.1. Study Design and Population

We conducted a matched case–control study to explore the association between clinical and lifestyle factors and bone parameters related to CFBD. The study included 32 people with CF and 70 healthy volunteers. Each individual with CF was individually matched with two healthy controls based on age (±5 years), sex, and body mass index (±3 kg/m^2^). In a limited number of cases, a third control was included to preserve the matching criteria when incomplete data were present in one of the initially selected controls, resulting in a variable matching ratio (1:2–1:3). All between-group analyses accounted for this individual matching strategy.

Participants were sequentially recruited at the Endocrinology Unit of University Hospital Clínico San Cecilio (Granada, Spain) between December 2023 and March 2025. Cases were referred from specialized CF outpatient clinics within several hospitals in Andalusia (Spain), including Hospital Universitario Reina Sofía (Córdoba), University Hospital of Jaén, Regional University Hospital of Málaga, University Hospital Virgen de las Nieves, and University Hospital Clínico San Cecilio of Granada.

Inclusion criteria for the CF group were: diagnosis of CF according to CFF consensus criteria [13], age ≥ 16 years, completion of pubertal development (Tanner stage V), stable clinical status for at least 1 month (no respiratory exacerbations requiring intravenous antibiotics and no body weight change >3%) and ability to provide written informed consent. Exclusion criteria for both groups included any metabolic bone disease or chronic illness affecting bone metabolism, chronic liver or kidney disease, cancer, and current use of hormone replacement therapy or osteoporosis treatment. Participants with artifacts preventing appropriate DXA evaluation of the region of interest were excluded according to the study exclusion criteria.

Healthy controls were recruited as apparently healthy volunteers from the hospital reference population. Controls were matched to patients with cystic fibrosis by sex, age, and body mass index (BMI). Individuals were excluded if they met any of the exclusion criteria applied to the CF group or had known conditions or treatments that could affect bone metabolism. Information on potential confounding factors related to bone health was collected for both groups, including smoking status, vitamin D supplementation, and physical activity. Physical activity was assessed using BPAQ. Serum vitamin D levels were also measured. These variables were considered in the analysis and are summarized in Table 1.

The study was approved by the Ethics Committee of University Hospital Clínico San Cecilio and conducted in accordance with the Declaration of Helsinki (Project ID: 0044-N-23). All participants gave written informed consent.

### 2.2. Clinical Assessments

In people with CF, clinical history included CFTR genotype, time since diagnosis, presence of bronchiectasis, exocrine pancreatic insufficiency, CFRD (cystic fibrosis-related diabetes) or dysglycemia, use of CFTR modulatortherapy (included in statistical analyses as a dichotomous variable: current use vs. no use), prolonged glucocorticoid use (>3 months), oral nutritional supplements (ONS) intake, number of pulmonary exacerbations in the previous year and history of fragility fractures. Fracture history was obtained through participant self-report during the clinical assessment and, whenever available, confirmed by review of medical records. Fragility fractures were defined as fractures occurring after low-energy trauma, such as a fall from standing height or less, and included major osteoporotic sites (vertebral, hip, wrist, and humerus). Only fractures occurring prior to the study assessment were recorded. Pulmonary function tests were performed following American Thoracic Society (ATS)/European Respiratory Society (ERS) guidelines [14] with forced expiratory volume in 1 s (FEV_1_) and forced vital capacity (FVC) by spirometry expressed as percentages of predicted values. Lifestyle factors included adherence to the Mediterranean diet (MedDiet), assessed with the validated 14-item PREDIMED questionnaire. The score ranges from 0 to 14 points, with higher scores indicating greater adherence; for analytical purposes, a score ≥9 was considered indicative of high adherence to the MedDiet [15] and physical activity level (evaluated with the Bone-specific Physical Activity Questionnaire or BPAQ) [16].

### 2.3. Body Composition, Muscle Strength, and Function

Anthropometric measurements included weight (kg) and height (m) measured with calibrated SECA equipment (SECA, Birmingham, UK). Body mass index (BMI) was calculated as weight (kg) divided by height squared (m^2^). Body composition was measured by DXA (Lunar Prodigy Advance, GE Healthcare, Chicago, IL, USA) with EnCore software (version 12.3; GE Healthcare, Chicago, IL, USA). Variables obtained included fat mass (FM) and fat-free mass (FFM). Fat-free mass index (FFMI) was calculated as FFM (kg)/height^2^. Low FFMI was defined according to ESPEN (European Society for Clinical Nutrition and Metabolism) criteria (FFMI < 15 kg/m^2^ in women and <17 kg/m^2^ in men) [17].

Muscle strength was assessed by handgrip dynamometry (Jamar® Hydraulic Hand Dynamometer, Asimow Engineering Co., Ltd. Santa Monica, CA, USA) following standardized procedures and defining low strength values below the 10th percentile of the Spanish reference population [18]. Functional exercise capacity was measured using the 6 min walk test according to ATS guidelines [19].

### 2.4. Laboratory Measurements

Venous blood samples were collected in the morning after an overnight fast. Serum aliquots were stored at −80 °C until analysis at the Clinical Analysis Unit of University Hospital Clínico San Cecilio (Granada, Spain). Biochemical tests included total calcium (mg/dL), phosphate (mg/dL), magnesium (mg/dL), creatinine (mg/dL), albumin (g/dL), prealbumin (mg/dL), fasting plasma glucose (mg/dL) and glycated hemoglobin (HbA1c, %), parathyroid hormone (PTH, pg/mL), 25-hydroxyvitamin D (ng/mL), C-terminal telopeptide of type I collagen (CTX, ng/mL), and procollagen type I N-terminal propeptide (P1NP, ng/mL) were measured using standard automated laboratory techniques. Similarly, alkaline phosphatase (ALP, IU/L) and bone-specific alkaline phosphatase (BSAP IU/L) were collected. Prothrombin time was also determined (%). Finally, inflammatory markers included high-sensitivity C-reactive protein (CRP, mg/L) and interleukin-6 (IL-6, pg/mL).

### 2.5. Bone Health Assessment

#### 2.5.1. Areal Bone Mineral Density

aBMD was measured at the LS (L1–L4), total hip (TH) and femoral neck (FN) using a Lunar Prodigy Advance densitometer (GE Healthcare, USA) following International Society for Clinical Densitometry (ISCD) recommendations [20].

DXA results were interpreted according to European Cystic Fibrosis Society guidelines. CF-related low BMD was defined as a Z-score ≤ –2.0, while normal BMD corresponded to Z-scores ≥ –1.0 [21].

#### 2.5.2. Trabecular Bone Score

TBS was derived from LS DXA scans (L1–L4) using TBS iNsight^®^ software (v3.0.2.0; Med-Imaps, Merignac, France). It provides an indirect estimate of trabecular microarchitecture based on pixel gray-level texture. Vertebrae with artifacts, fractures, or degenerative changes were excluded, following manufacturer recommendations [22]. Participants with artifacts preventing appropriate DXA evaluation were excluded according to the study exclusion criteria; therefore, no additional exclusions were required for the TBS analysis.

TBS categories used in the general population were applied: ≥1.31 indicating normal microarchitecture, 1.23–1.31 partially degraded, and ≤1.23 degraded [22]. No cystic fibrosis-specific cutoffs are currently established.

#### 2.5.3. 3D-DXA Modeling

3D modeling of the proximal femur was performed using 3D-Shaper^®^ software (version 2.14.0; Galgo Medical, Barcelona, Spain) applied to standard DXA hip scans. This validated technique reconstructs a 3D model from two-dimensional DXA images to estimate cortical surface BMD (sBMD, mg/cm^2^) and trabecular vBMD (mg/cm^3^) [5].

Both parameters are essential for bone strength. Cortical sBMD reflects mineralization of the cortical envelope and resistance to bending/torsion, whereas trabecular vBMD reflects mineral content and capacity to withstand compressive loads. As no clinical cutoffs exist, values were interpreted comparatively between groups and alongside aBMD and TBS.

Z-scores and T-scores for 3D-DXA parameters were calculated using the age- and sex-adjusted normative reference database derived from a Spanish population, as implemented in the 3D-Shaper^®^ software.

Post hoc 3D-DXA visualizations were generated after completion of the statistical analyses, once between-group differences had already been identified. The vendor was aware of group allocation at the time of image generation; however, their role was strictly limited to technical assistance with image processing and visualization. The vendor was not involved in study design, data analysis, statistical modeling, or interpretation of results.

### 2.6. Statistical Analysis

Categorical variables were summarized as counts and percentages. Continuous variables were summarized as mean (standard deviation, SD) if approximately normally distributed or as median (interquartile range, IQR) otherwise. Distributional normality was assessed using the Shapiro–Wilk test. Given the individually matched design (26 sets matched 1:2 and 6 sets matched 1:3), group comparisons were performed using linear mixed-effects models for continuous variables and generalized linear mixed models (binomial family) for categorical variables, both incorporating the matched set as a random intercept. Degrees of freedom and *p*-values for linear mixed-effects models were estimated using the Kenward–Roger approximation. Correlations between bone parameters and clinical measures in people with CF were estimated using Pearson or Spearman coefficients, as appropriate.

To identify factors associated with bone parameters, multivariable linear regression models were fitted within the CF group (*n* = 32). Candidate predictors for each outcome were selected a priori based on established risk factors for CF bone disease and biological plausibility. Final models were obtained using a genetic algorithm for automatic model selection [23] with the small-sample corrected Akaike information criterion (AICc) as the optimization target. Model assumptions were evaluated through statistical tests and graphical diagnostics, and influence diagnostics (Cook’s distance) were examined to ensure that no individual observation disproportionately affected the results. Missing data were minimal (<5% for all variables) and complete-case analysis was used. All tests were two-sided with α = 0.05. Analyses were conducted in R version 4.5.1.

## 3. Results

### 3.1. Study Population Characteristics

Study population characteristics are shown in Table 1. Groups were well balanced for sex (*p* = 0.810) and age (*p* = 0.951); however, despite individual matching by BMI (±3 kg/m^2^), a statistically significant difference persisted between groups (CF: 22.4 vs. controls: 23.5 kg/m^2^; *p* = 0.003). Accordingly, all subsequent between-group comparisons were additionally adjusted for BMI as a fixed-effect covariate in the mixed-effects models.

**Table 1:** Baseline demographic, clinical, and biochemical characteristics of the study population.

	CF *N* = 32 ^1^	Controls *N* = 70 ^1^	*p*-Value ^2^
DEMOGRAPHIC DATA			
Gender			0.810
Male	20 (62.5%)	42 (60.0%)	
Female	12 (37.5%)	28 (40.0%)	
Age (years)	33.5 (24.5, 45.5)	31.0 (27.0, 43.0)	0.951
BMI (kg/m^2^)	22.4 (21.2, 23.91)	23.5 (21.3, 25.4)	0.003
Fragility fractures prevalence	7 (22.2%)	-	
Vertebral fragility fractures	2 (28.6%)		
Nonvertebral fragility fractures	5 (71.4%)		
CF-related variables			
Genotype			
Heterozygous F508del	15 (46.9%)	-	
Homozygous F508del	8 (25.0%)	-	
Others	9 (28.1%)	-	
Disease duration (years)	26.0 (18.0, 34.0)	-	
Bronchiectasis	29 (90.6%)	-	
FEV_1_%	73.9 (55, 88)	-	
FVC%	84.5 (69.5, 96.5)	-	
Total exacerbations per year	1.0 (0.0, 2.0)	-	
Exocrine pancreatic insufficiency	24 (75.0%)	-	
CFRD	7 (21.9%)	-	
Dysglycemia	6 (19.4%)	-	
CFTR modulator therapy	22 (71.0%)	-	
ONS intake	14 (44%)	-	
Corticosteroids > 3 months	6 (19.4%)	-	
BODY COMPOSITION by DXA			
FM (kg)	16.0 (12.3, 21.5)	16.5 (13.8, 21.7)	0.339
FFM (Kg)	47.8 (38.9, 57.5)	54.0 (40.6, 60.4)	0.211
FFMI (kg/m^2^)	16.5 (14.7, 18.5)	17.0 (14.9, 19.2)	0.323
Low FFMI	12 (37.5%)	26 (37.7%)	0.986
MUSCLE STRENGTH			
Handgrip dynamometry (Kg)	31 (25, 42.8)	36.2 (34.5, 38)	0.308
Low strength (percentile < 10)	11 (34.4%)	4 (6.1%)	<0.001 *
PHYSICAL PERFORMANCE			
6-Minute Walk Test	525.0 (486, 564.0)	528.3 (495.0, 600.0)	0.607
LIFESTYLE			
Predimed questionnaire (points)	8.5 (7.0, 10.0)	10.0 (9.0, 11.0)	<0.001 *
Mediterranean diet adherence	16 (50.0%)	56 (81.2%)	0.001 *
BPAQ	17.5 (0.8, 47.0)	20.2 (4.9, 61.8)	0.285
BIOCHEMICALS PARAMETERS			
Glucose (mg/dL)	86 (78, 93.5)	83 (77, 87)	0.046 *
HbA1c (%)	5.5 (5.4, 6)	5.3 (5.1, 5.5)	<0.001 *
Creatinine (mg/dL)	0.7 (0.6, 0.8)	0.9 (0.7, 1)	0.526
Albumin (g/dL)	4.5 (4.2, 4.8)	4.7 (4.6, 4.9)	0.005 *
Prealbumin (mg/dL)	28 (23.6, 31.0)	26.5 (24.1, 30.5)	0.695
PTH (pg/mL)	68 (60, 80)	56 (44, 75)	0.013 *
Calcium (mg/dL)	9.4 (9.1, 9.6)	9.4 (9.2, 9.7)	0.46
Phosphate (mg/dL)	3.3 (2.9, 3.6)	3.4 (3.0, 3.7)	0.392
25-hydroxyvitamin D (ng/mL)	32.9 (21.3, 39.4)	26.3 (19.7, 31.1)	0.017 *
Magnesium (mg/dL)	2 (1.9, 2.1)	2.1 (2.0, 2.2)	0.041 *
ALP (IU/L)	114.0 (82.0, 132.0)	68.5 (56.0, 85.0)	<0.001 *
BSAP (IU/L)	18 (13, 23)	13 (9, 16)	<0.001 *
P1NP (ng/mL)	76.7 (55.6, 87.9)	69.7 (60.1, 85)	0.87
CTX (ng/mL)	0.4 (0.3, 0.6)	0.4 (0.4, 0.6)	0.435
CRP (mg/L)	2.3 (0.7, 9.4)	0.8 (0.4, 1.2)	<0.001 *
IL-6 (pg/mL)	3 (1.9, 6.6)	1.8 (1.4, 2.4)	<0.001 *
Prothrombin time (%)	99.5 (91, 107)	99 (92, 105)	0.535

^1^ n (%); Median (Q1, Q3); ^2^ Continuous variables were compared using linear mixed-effects models and categorical variables using generalized linear mixed models, both with the matched set as a random intercept to account for the individually matched design. Due to residual imbalance in BMI between groups despite matching, all models were additionally adjusted for BMI as a fixed-effect covariate. Abbreviations: CF: Cystic Fibrosis; BMI: Body mass index; FVC: forced vital capacity; FEV_1_: forced expiratory volume in one second; CFRD: Cystic fibrosis related diabetes; CFTR: Cystic Fibrosis Transmembrane Conductance Regulator; ONS: oral nutritional supplements; FM: fat mass; FFM fat-free mass; FFMI: fat-free mass index; BPAQ: Bone-specific Physical Activity Questionnaire; HbA1c: glycated hemoglobin; PTH: Parathyroid hormone; ALP: alkaline phosphatase; BSAP: bone-specific alkaline phosphatase; P1NP: Procollagen type I N-terminal propeptide; CTX: terminal telopeptide of type I collagen; CRP: C-reactive protein; IL-6: interleukin-6. * Significant differences between groups (*p* ≤ 0.05)

Most people with CF carried at least one F508del mutation and more than two-thirds were receiving CFTR modulator therapy. Pulmonary function was moderately impaired (median FEV_1_ 73.9% predicted), bronchiectasis was almost universal and exocrine pancreatic insufficiency and CFRD were common. A large proportion of CF adults were also receiving chronic inhaled corticosteroid therapy. The prevalence of fragility fractures was higher in people with CF.

Overall, FM and FFM did not differ between groups. Median handgrip strength values were also comparable between CF participants and controls. However, the proportion of individuals with low handgrip strength was higher in the CF group, affecting approximately one third of people compared with only 6% of controls (*p* < 0.05). In contrast, performance in the six-minute walk test did not differ between groups.

Regarding lifestyle, people with CF showed lower adherence to the MedDiet with lower PREDIMED scores (*p* = 0.001). Physical activity levels were similar between groups.

People with CF presented higher HbA1c and 25-hydroxyvitamin D levels, increased bone turnover markers (PTH, ALP, and BSAP) and elevated inflammatory markers (CRP and IL-6), together with lower serum albumin (all *p* < 0.05).

### 3.2. Bone Structural Parameters in Study Population

Bone structural parameters are summarized in Table 2. The prevalence of low areal bone mass was higher in the CF group than in controls (28.1% vs. 5.7%, *p* < 0.001). TBS categories also differed: while most controls showed normal values, a higher proportion of CF people presented partially degraded or degraded microarchitecture (18.8% vs. 4.3% or 12.5% vs. 0%; *p* = 0.002).

**Figure 1:**
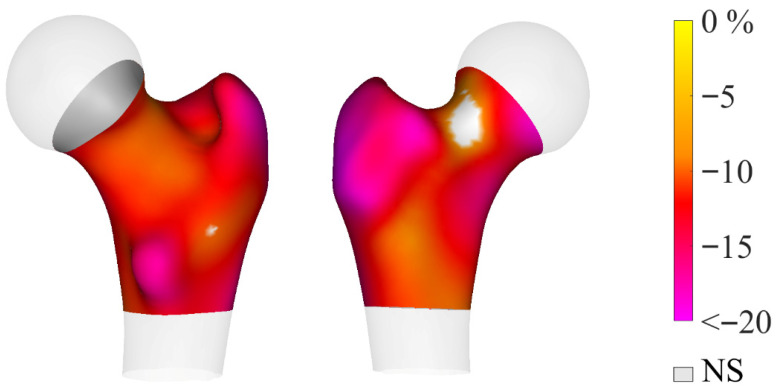
Three-dimensional visualization showing the anatomical distribution of percentage differences in cortical sBMD between cases and controls (*p* < 0.05). Non-significant (NS) differences are shown in gray. The map illustrates regional variations in cortical surface bone mineral density across the proximal femur.

**Figure 2:**
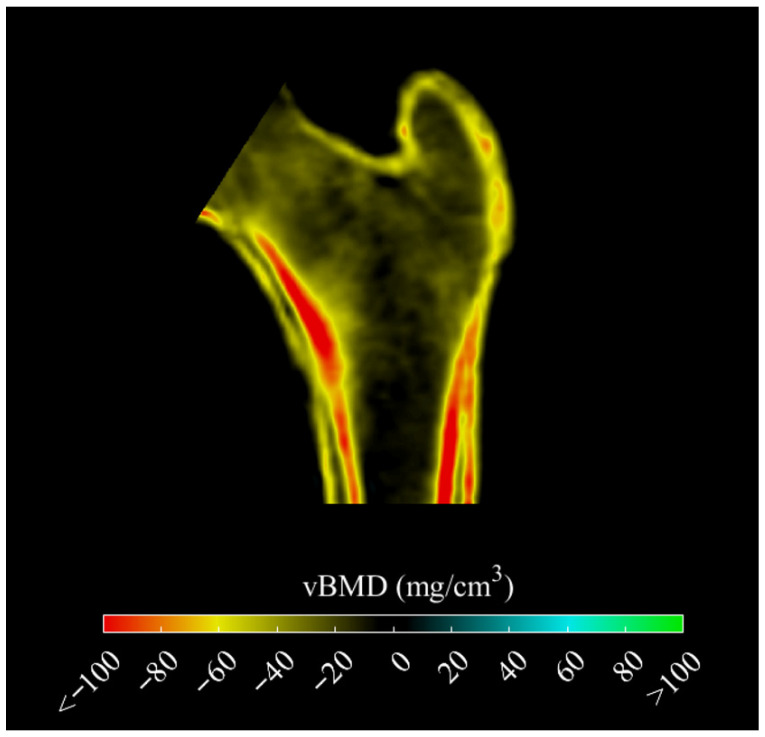
Mid-coronal cross-section showing the anatomical distribution of differences in cortical and trabecular vBMD (mg/cm^3^) between cases and controls. Abbreviation: vBMD: volumetric bone mineral density.

Table 2 shows differences in aBMD, TBS, and 3D-DXA parameters between groups. Sex-stratified descriptive results for these parameters are provided in Supplementary Table S3. aBMD was lower in the CF group compared with controls at all sites: FN, TH, and LS (all *p* < 0.05). Similarly, TBS values were lower in the CF group (*p* < 0.001). Cortical sBMD (Figure 1) and trabecular vBMD (Figure 2) by 3D-DXA were also lower in people with CF (all *p* < 0.005).

### 3.3. Correlations Between Bone Parameters by 3D-DXA and TBS, aBMD, Lifestyle, Biochemicals Markers and Muscle Status in Cystic Fibrosis Population

Correlations are reported in Supplementary Tables S1 and S2. Trabecular vBMD and cortical sBMD showed strong positive correlations with aBMD at the FN and TH (r > 0.79; *p* < 0.001). Trabecular vBMD was also positively correlated with LS aBMD and TBS (r > 0.48; *p* = 0.005) and inversely correlated with FM measured by DXA (r = −0.40; *p* = 0.024). Cortical sBMD was positively correlated with LS aBMD, respiratory function (FEV_1_ and FVC% predicted), and FFM measured by DXA (all r > 0.36; *p* < 0.05). No significant association was observed between cortical sBMD and TBS (*p* > 0.05). TBS showed a positive correlation with HbA1c (r = 0.458; *p* < 0.05). Predictors of bone parameters (aBMD, TBS, femoral 3D-DXA) in people with cystic fibrosis.

Associations between clinical variables and bone parameters (aBMD, TBS, and femoral 3D-DXA measures) in adults with CF are presented in Table 3. In the multivariable regression analyses, adherence to the MedDiet was associated with several femoral and microarchitectural bone parameters, including FN and TH aBMD, TBS, trabecular vBMD, and cortical sBMD (*p* ≤ 0.01), but not with LS aBMD (*p* = 0.739). Female sex was also associated with several bone outcomes including FN and TH aBMD, TBS, trabecular vBMD, and cortical sBMD (*p* ≤ 0.02), but not with LS aBMD (*p* = 0.343). Other variables associated with bone parameters included FFM (β = 0.007 to 1.7, *p* ≤ 0.045), FM (β = −0.010 to −0.015, *p* ≤ 0.01), prolonged corticosteroid use (β = −0.16 to −0.34, *p* ≤ 0.02), ONS intake (β = −0.10 to −0.18, *p* ≤ 0.02), FEV_1_ (β = 0.003, *p* = 0.021), disease duration (β = 0.003, *p* = 0.039), and P1NP levels (β = 0.002 to 0.60, *p* ≤ 0.044).

**Figure 3:**
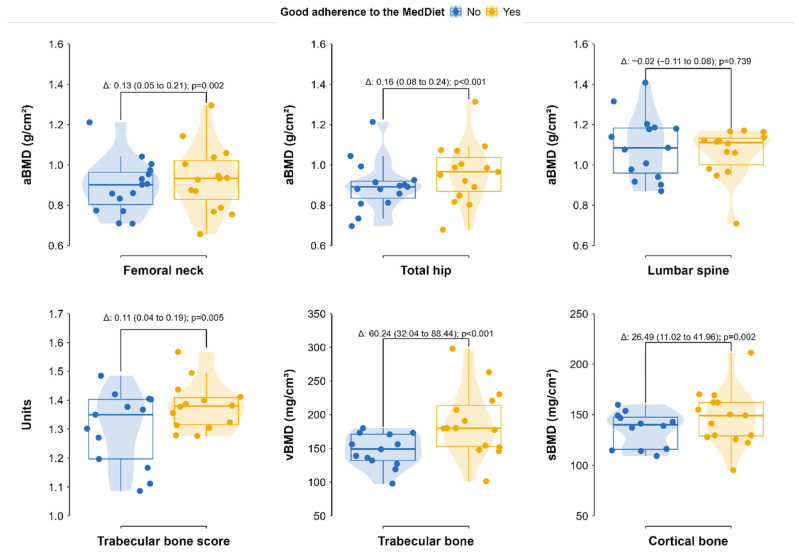
Bone parameters according to Mediterranean diet adherence in adults with CF. Mean values (95% CIs) for femoral neck, total hip, and lumbar spine aBMD, trabecular bone score, trabecular vBMD, and cortical sBMD are shown for people with (yellow) and without (blue) good adherence to the Mediterranean diet. Abbreviations: BMD: bone mineral density; vBMD: volumetric bone mineral density; and sBMD: surface bone mineral density.

As illustrated in Figure 3, people with higher adherence to the MedDiet showed higher FN and TH aBMD, TBS, trabecular vBMD, and cortical sBMD compared with those with lower adherence (all *p* ≤ 0.005), whereas no differences were observed at the LS (*p* = 0.739).

## 4. Discussion

This study simultaneously analyzes bone structure alterations using three different tools: aBMD, vBMD, and TBS. The results show marked structural deficits and identify several clinical and lifestyle factors related to bone parameters, including adherence to the MedDiet pattern. Importantly, this is the first study to provide an integrated assessment of bone health in people with CF using 3D-DXA and TBS. This approach enables a more comprehensive analysis of cortical and trabecular compartments and may reveal structural abnormalities that remain undetected by conventional DXA.

Despite improvements in clinical management and the use of CFTR modulator therapy, the prevalence of CFBD and fragility fractures remained high in our cohort. Our findings are consistent with previous reports describing CFBD as a common complication among adults, with low BMD and an increased risk of fractures documented in 20% to 60% of people. [1]. Likewise, adults with CF showed significantly lower aBMD than controls [9]. Putman et al. even demonstrated that bone loss accelerates in adulthood, meaning that many people with CF may reach menopause or old age with suboptimal bone mass [24]. These observations highlight the need for bone monitoring and specific strategies to preserve bone integrity in adults with CF.

Additionally, our results revealed impaired trabecular microarchitecture by TBS in adults with CF. Only one previous study had examined TBS in this population and also found lower values, even after adjusting for aBMD [6]. This finding may suggest that microarchitectural damage can occur independently of bone mass. In addition, TBS has been validated as an adjunct to BMD for improving fracture risk prediction [25]. Therefore, integrating TBS into routine assessments could enhance bone risk stratification and facilitate earlier identification of bone fragility in adults with CF.

Our 3D-DXA analysis identified impairments in both cortical and trabecular compartments in people with CF compared with controls, indicating compromised bone strength. Our results were aligned with previous HR-pQCT and pQCT studies [7,26,27]. Importantly, Kelly et al. showed trabecular and cortical abnormalities even in people with normal aBMD values [7], suggesting that structural impairment may precede changes detectable by aBMD. However, pQCT is a research tool. Alternatively, 3D-DXA is emerging as a practical tool that provides bone volume estimates, maintaining the accessibility, low radiation exposure, and cost-effectiveness of conventional DXA [28]. In fact, the strong agreement between measurements derived from 3D DXA and those obtained using pQCT supports its usefulness as a substitute for more complex 3D imaging techniques [5,29]. Furthermore, 3D-DXA studies demonstrated its ability to detect relevant structural alterations in other secondary osteoporosis conditions [30]. Overall, the 3D-DXA alterations observed likely reflect meaningful skeletal deterioration, supporting potential usefulness for characterizing bone structure in adults with CF.

On the other hand, we identified several determinants independently associated with bone structural parameters in people with CF. Female sex and greater adherence to the MedDiet were associated with several bone outcomes in the regression analyses, although this association was not observed for LS aBMD. In the general population, MedDiet adherence has been associated with higher BMD [31]. Although no studies have specifically evaluated this dietary pattern in people with CF, its basic principles are consistent with current recommendations for optimizing bone health in this population [32,33]. Furthermore, the relationship between sex and bone health in CF remains poorly understood. Although results are heterogeneous [34], evidence suggests that men with CF are at greater risk of poorer bone quality, likely due to more severe lung disease, lower FFM, and hormonal alterations [7,34,35,36]. Also, people with CF who received prolonged treatment with corticosteroids showed lower aBMD and reduced trabecular and cortical parameters, consistent with the well-described adverse skeletal effects of glucocorticoid [11]. Similarly, higher FFM has been consistently associated with better bone outcomes. In contrast, greater FM and the use for ONS were linked to lower bone structural parameters [7,9,37,38]. However, this finding should be interpreted with caution, as the prescription of ONS in clinical practice often reflects underlying disease severity or nutritional risk rather than a direct causal relationship with bone outcomes (confounding by indication). Pulmonary function (FEV_1_ and FVC) is a key prognostic determinant in people with CF and is closely intertwined with bone health [7,39]. In our cohort, FEV_1_ and FVC were correlated positively with cortical sBMD and TBS, and FEV_1_ was independently associated with LS aBMD. Interestingly, disease duration was positively associated with TBS. This unexpected finding may reflect a survival bias, whereby individuals with longer disease duration represent a more stable and nutritionally adequate subgroup, or it may indicate the cumulative impact of improvements in CF care over recent decades. The progressive implementation of structured multidisciplinary teams and the widespread adoption of CFTR modulator therapy may contribute to better preservation of bone health in adults with CF [8]. Vitamin D deficiency is frequently reported in CF and has been linked to lower BMD and increased fracture risk [10]. In our cohort, higher 25-hydroxyvitamin D concentrations were independently predicted FN aBMD. Notably, 25-hydroxyvitamin D levels were higher in the CF group than in controls, reflecting that most adults with CF were receiving supplementation (88%).

In our cohort, adults with CF showed higher levels of PTH, ALP and BSAP, consistent with increased bone turnover. These findings are consistent with previous studies reporting alterations in bone metabolism in CF. Aris et al. described elevated PTH levels together with increased bone turnover markers in adults with CF [40]. In CF, these alterations have been attributed to multiple mechanisms, including pancreatic insufficiency–related malabsorption of calcium and fat-soluble vitamins, chronic systemic inflammation and endocrine disturbances, all of which may contribute to dysregulated bone remodeling and skeletal deterioration [41].

This study has several strengths and some limitations. Strengths include a large, well-characterized cohort of adults with CF matched by age, sex, and BMI to healthy controls, ensuring robust comparisons. Despite matching for BMI—an important confounder in bone assessment—significant differences in bone structural parameters persisted, while FFM remained comparable between groups. This suggests that the bone deterioration observed in CF cannot be explained solely by differences in body composition. Major potential confounders—including CFTR genotype, inflammatory markers, and corticosteroid exposure—were recorded and incorporated into the analyses. The combined use of 3D-DXA, TBS, and conventional DXA allowed a multidimensional evaluation of bone status, providing novel insight into the structural deterioration associated with CFBD. However, the observational design precludes causal inference. Furthermore, the study design does not allow us to infer that 3D or TBS improve fracture risk estimation in people with CF. In addition, the relatively small sample size of the CF group may limit multivariate regression estimates; therefore, these findings should be interpreted with caution and considered exploratory. In addition, the study cohort consisted of clinically stable adults without oxygen therapy and with relatively preserved lung function (median FEV_1_ ≈ 74%), which mainly reflects individuals with mild-to-moderate disease severity. Therefore, caution is warranted when extrapolating these findings to patients with more advanced CF.

## 5. Conclusions

The prevalence of CFBD remains high in adults, highlighting the importance of comprehensive bone assessment in this population. The combined use of DXA, 3D-DXA and TBS provided complementary information: conventional DXA identified reductions in aBMD, 3D-DXA suggested compartment-specific cortical and trabecular alterations, and TBS indicated microarchitectural impairment that may not be detected by conventional DXA alone. Several clinical and nutritional variables—including adherence to the MedDiet, sex, corticosteroid exposure, and pulmonary function were associated with bone parameters, reflecting the multifactorial nature of bone fragility in CF. These findings support the potential value of an integrated evaluation approach and emphasize the importance of optimizing nutritional status, lifestyle factors, and clinical management to improve early detection and prevention of bone deterioration in adults with CF. Further studies are needed to confirm these preliminary results.

## Figures and Tables

**Table 2:** Differences in areal bone mineral density (aBMD), trabecular bone score (TBS), and 3D-DXA parameters between study groups.

Characteristic	CF *N* = 32 ^1^	Controls *N* = 70 ^1^	Δ ^2^	95% CI	*p*-Value
aBMD by 2D-DXA					
FN BMD (g/cm^2^)	0.92 (0.15)	1.01 (0.14)	−0.08	−0.14, −0.04	<0.002 *
Z-score	−0.61 (1.01)	−0.01 (0.89)	−0.56	−0.97, −0.23	0.004 *
TH BMD (g/cm^2^)	0.94 (0.14)	1.03 (0.15)	−0.08	−0.15, −0.05	<0.003 *
Z-score	−0.68 (1.01)	−0.06 (0.98)	−0.57	−1.01, −0.26	0.004 *
LS BMD (g/cm^2^)	1.08 (0.14)	1.20 (0.14)	−0.11	−0.17, −0.06	<0.001 *
Z-score	−0.66 (1.17)	0.07 (1.06)	−0.72	−1.16, −0.3	0.002 *
TBS					
LS TBS	1.35 (0.11)	1.43 (0.08)	−0.08	−0.11, −0.05	<0.001 *
3D-DXA					
Cortical sBMD (mg/cm^2^)	145.09 (24.78)	166.23 (22.98)	18.1	−30.1, −13.4	<0.001 *
Z-score	−0.95 (1.08)	0.01 (0.99)	−0.85	−1.35, −0.60	<0.001 *
Trabecular vBMD (mg/cm^3^)	172.50 (47.47)	196.17 (47.37)	20.9	−40.3, −6.83	0.020 *
Z-score	−0.49 (1.05)	0.07 (1.11)	−0.48	−0.98, −0.13	0.033 *

^1^ Mean (SD); ^2^ Between-group differences were estimated using linear mixed-effects models with the matched set as a random intercept and BMI as a fixed-effect covariate to account for residual imbalance despite matching. Abbreviations: Δ, Difference; CF: cystic fibrosis; CI: confidence interval; aBMD: areal bone mineral density; BMD: bone mineral density; FN: femoral neck, TH: total hip, LS: lumbar spine; TBS: trabecular bone score; sBMD: surface bone mineral density; vBMD: volumetric bone mineral density. * Significant differences between groups (*p* ≤ 0.05)

**Table 3:** Multiple regression models for bone parameters (aBMD, TBS, and femoral 3D-DXA) in people with cystic fibrosis.

Predictors	β	95% CI	*p*-Value
FN aBMD, aR^2^ = 0.63			
Intercept	0.5296	0.1966, 0.8625	0.003
ONS intake	−0.1171	−0.2041, 0.0301	**0.011**
Female sex	0.1934	0.0744, 0.3124	**0.003**
Corticosteroids > 3 months	−0.1567	−0.2506, −0.0629	**0.002**
25-hydroxyvitamin D levels	0.0036	0.0005, 0.0067	**0.027**
FFM	0.0085	0.0038, 0.0132	**0.001**
FM	−0.0146	−0.0213, −0.0080	**<0.001**
Mediterranean diet adherence	0.1446	0.0649, 0.2243	**0.001**
FEV_1_ (%)	0.0004	−0.0013, 0.0022	0.620
TH aBMD, aR^2^ = 0.58			
Intercept	0.4824	0.1507, 0.8140	0.007
ONS intake	−0.1046	−0.1913, −0.0180	**0.020**
Female sex	0.1740	0.0554, 0.2925	**0.006**
Corticosteroids > 3 months	−0.1638	−0.2573, −0.0703	**0.002**
25-hydroxyvitamin D levels	0.0025	−0.0006, 0.0057	0.104
FFM	0.0076	0.0029, 0.0122	**0.003**
FM	−0.0103	−0.0170, −0.0037	**0.004**
Mediterranean diet adherence	0.1695	0.0901, 0.2488	**<0.001**
FEV_1_ (%)	0.0013	−0.0005, 0.0031	0.140
LS aBMD, aR^2^ = 0.40			
Intercept	1.1260	0.8800, 1.3720	<0.001
Molecular treatment	−0.1005	−0.2036, 0.0026	0.056
ONS intake	−0.1833	−0.2924, −0.0741	**0.002**
Female sex	0.0517	−0.0596, 0.1630	0.343
Corticosteroids > 3 months	−0.0744	−0.1969, 0.0481	0.219
FM	−0.0136	−0.0235, −0.0037	**0.010**
FEV_1_ (%)	0.0026	0.0004, 0.0049	**0.021**
Age	0.0007	−0.0041, 0.0055	0.749
25-hydroxyvitamin D levels	0.0032	−0.0009, 0.0073	0.115
Mediterranean diet adherence	−0.0156	−0.1118, 0.0806	0.739
TBS, aR^2^ = 0.45			
Intercept	0.9748	0.7876, 1.1620	<0.001
CFTR modulators therapy	−0.0252	−0.1121, 0.0618	0.549
Mediterranean diet adherence	0.1148	0.0374, 0.1922	**0.006**
FEV_1_ (%)	0.0010	−0.0012, 0.0033	0.357
BSAP	0.0037	−0.0001, 0.0075	0.055
Time from diagnosis	0.0033	0.0002, 0.0064	**0.039**
Female sex	0.1154	0.0208, 0.2099	**0.020**
Corticosteroids > 3 months	−0.0775	−0.1903, 0.0353	0.165
P1NP	0.0017	0.0002, 0.0031	**0.027**
Trabecular vBMD, aR^2^ = 0.54			
Intercept	13.879	−135.62, 163.38	0.849
Age	−0.4319	−1.7813, 0.9175	0.513
Female sex	59.691	15.763, 103.62	**0.010**
Mediterranean diet adherence	60.244	32.045, 88.444	**<0.001**
P1NP	0.5961	0.0165, 1.1757	**0.044**
Corticosteroids > 3 months	−38.319	−69.866, −6.7711	**0.020**
FFM	1.6992	0.0404, 3.3580	**0.045**
Cortical sBMD, aR^2^ = 0.47			
Intercept	47.206	−11.673, 106.08	0.110
Time from diagnosis	−0.0716	−0.5657, 0.4226	0.766
Female sex	27.651	4.8892, 50.414	**0.020**
Mediterranean diet adherence	26.493	11.023, 41.964	**0.002**
Corticosteroids > 3 months	−34.226	−53.171, −15.280	**0.001**
FFM	1.3240	0.4039, 2.2442	**0.007**
25-hydroxyvitamin D levels	0.4312	−0.1635, 1.0259	0.146

Boldface denotes significant values (*p* < 0.05). Abbreviations: β: regression coefficient; aR^2^: adjusted R-squared; FN: femoral neck; aBMD: areal bone mineral density; TH: total hip; LS: lumbar spine; FEV_1_: Forced expiratory volume in 1 s; P1NP: procollagen type I N-terminal propeptide; vBMD: volumetric bone mineral density; TBS: trabecular bone score; SBMD: surface bone mineral density.

## Data Availability

The data presented in this study are available on request from the corresponding author.

## Supplementary Materials

The following supporting information can be downloaded at: https://www.mdpi.com/article/10.3390/jcm15062366/s1, Table S1: Correlations between trabecular bone and TBS, aBMD, lifestyle, biochemicals markers and muscle status in Cystic Fibrosis population. Table S2: Correlations between cortical bone and TBS, aBMD, lifestyle, biochemicals markers and muscle status in Cystic Fibrosis population. Table S3: Sex-stratified descriptive analysis of bone parameters.
