# Supplementary material for: Bone Structural Parameters in Adults with Cystic Fibrosis: Contribution of Adherence to the Mediterranean Diet
Source: J Clin Med. 2026 Mar 19;15(6):2366. doi: 10.3390/jcm15062366 (PMC13027349; doi:10.3390/jcm15062366)
Supplement: Supplementary file 1 [file jcm-15-02366-s001.zip › Table S1.pdf]

Supplementary Material

Table S1. Correlations between trabecular bone and TBS, aBMD, lifestyle, biochemicals markers and muscle status in Cystic Fibrosis population

Parameter 1	Parameter 2	r	Lower CI	Upper CI	*p value*
Trabecular vBMD	**TBS**	**0.489**	**0.152**	**0.724**	**0.005**
	**NF BMD**	**0.793**	**0.603**	**0.898**	**<0.001**
	**TH BMD**	**0.828**	**0.664**	**0.916**	**<0.001**
	**LS BMD**	**0.495**	**0.160**	**0.728**	**0.005**
	Predimed questionnaire	0.227	-0.149	0.546	0.220
	IPAQ- SF	0.037	-0.344	0.408	0.847
	Handgrip dynamometry	0.195	-0.182	0.522	0.293
	**FM**	**-0.405**	**-0.670**	**-0.048**	**0.024**
	FFM	0.122	-0.253	0.465	0.514
	FFMI	0.183	-0.194	0.512	0.325
	CRP	-0.040	-0.404	0.334	0.832
	IL-6	0.080	-0.313	0.449	0.687
	FEV1 (%)	0.016	-0.350	0.377	0.934
	FVC (%)	0.117	-0.258	0.461	0.530
	**PNP1**	**0.344**	**-0.037**	**0.638**	**0.067**
	CTX	0.106	-0.281	0.464	0.584
	ALP	0.240	-0.142	0.561	0.201
	BSAP	0.222	-0.169	0.552	0.248
	PTH	0.050	-0.333	0.418	0.797
	25-hydroxyvitamin D	-0.146	-0.489	0.237	0.442

The p-values between the different associations were estimated using Pearson or Spearman coefficients, as appropriate. Abbreviations: vBMD: volumetric bone mineral density; CI: confidence interval; TBS: Trabecular bone score; BMD: bone mineral density; FN: femoral neck, TH: total hip, LS: lumbar spine; IPAQ-SF: International Physical Activity Questionnaire – short form; FM: fat mass; FFM fat free mass; FFMI: fat free mass index; CRP: C-reactive protein; IL-6: interleukin-6; FEV1: forced expiratory volume in one second; FVC: forced vital capacity; P1NP: Procollagen type I N-terminal propeptide; CTX: -terminal telopeptide of type I collagen; ; ALP: alkaline phosphatase; BSAP:bone-specific alkaline phosphatase; PTH: Parathyroid hormone.
