# Supplementary material for: Bone Structural Parameters in Adults with Cystic Fibrosis: Contribution of Adherence to the Mediterranean Diet
Source: J Clin Med. 2026 Mar 19;15(6):2366. doi: 10.3390/jcm15062366 (PMC13027349; doi:10.3390/jcm15062366)
Supplement: Supplementary file 1 [file jcm-15-02366-s001.zip › Table S2.pdf]

Supplementary Material

Table S2. Correlations between cortical bone and TBS, aBMD, lifestyle, biochemicals markers and muscle status in Cystic Fibrosis population

Parameter 1	Parameter 2	r	Lower CI	Upper CI	*p value*
Cortical sBMD	TBS	0.275	-0.099	0.581	0.135
	**NF BMD**	**0.796**	**0.608**	**0.899**	**<0.001**
	**TH BMD**	**0.932**	**0.859**	**0.968**	**<0.001**
	**LS BMD**	**0.540**	**0.219**	**0.755**	**0.002**
	Predimed questionnaire	0.023	-0.344	0.383	0.903
	IPAQ- SF	0.110	-0.278	0.467	0.569
	Handgrip dynamometry	0.270	-0.105	0.577	0.143
	FM	-0.187	-0.516	0.190	0.314
	**FFM**	**0.399**	**0.041**	**0.666**	**0.026**
	FFMI	0.334	-0.034	0.622	0.067
	CRP	-0.143	-0.487	0.240	0.451
	IL-6	-0.033	-0.411	0.354	0.867
	**FEV1 (%)**	**0.362**	**-0.002**	**0.642**	**0.045**
	**FVC (%)**	**0.454**	**0.107**	**0.702**	**0.010**
	PNP1	-0.003	-0.379	0.374	0.988
	CTX	-0.115	-0.471	0.273	0.554
	ALP	-0.018	-0.386	0.354	0.923
	BSAP	-0.149	-0.497	0.241	0.442
	PTH	0.032	-0.349	0.403	0.871
	25-hydroxyvitamin D	0.161	-0.222	0.501	0.395

The p-values between the different associations were estimated using Pearson or Spearman coefficients, as appropriate. Abbreviations: sBMD: surface bone mineral density; CI: confidence interval; TBS: Trabecular bone score; BMD: bone mineral density; FN: femoral neck, TH: total hip, LS: lumbar spine; IPAQ-SF: International Physical Activity Questionnaire – short form; FM: fat mass; FFM fat free mass; FFMI: fat free mass index; CRP: C-reactive protein; IL-6: interleukin-6; FEV1: forced expiratory volume in one second; FVC: forced vital capacity; P1NP: Procollagen type I N-terminal propeptide; CTX: -terminal telopeptide of type I collagen; ; ALP: alkaline phosphatase; BSAP:bone-specific alkaline phosphatase; PTH: Parathyroid hormone.
