# Supplementary material for: Bone Structural Parameters in Adults with Cystic Fibrosis: Contribution of Adherence to the Mediterranean Diet
Source: J Clin Med. 2026 Mar 19;15(6):2366. doi: 10.3390/jcm15062366 (PMC13027349; doi:10.3390/jcm15062366)
Supplement: Supplementary file 1 [file jcm-15-02366-s001.zip › Table S3.pdf]

Supplementary Material

Table S3. Sex-stratified descriptive analysis of bone parameters

**Characteristic**	**Cases**		**Controls**	
	**Male**<br>N = 20[1]	**Female**<br>N = 12[1]	**Male**<br>N = 42[1]	**Female**<br>N = 28[1]
BMD Femoral neck	0.94 (0.13)	0.89 (0.18)	1.04 (0.14)	0.96 (0.12)
T-score Femoral neck	-1.04 (1.03)	-0.88 (1.48)	-0.21 (1.13)	-0.20 (0.99)
Z-score Femoral neck	-0.69 (0.78)	-0.48 (1.34)	-0.05 (0.88)	0.05 (0.92)
BMD Total Hip	0.95 (0.12)	0.91 (0.17)	1.07 (0.15)	0.97 (0.12)
T-score Total Hip	-1.08 (0.97)	-0.86 (1.42)	-0.16 (1.21)	-0.23 (0.98)
Z-score Total Hip	-0.77 (0.83)	-0.53 (1.28)	-0.07 (1.01)	-0.04 (0.94)
BMD Lumbar spine	1.09 (0.14)	1.07 (0.13)	1.22 (0.13)	1.17 (0.14)
T-score Lumbar spine	-1.07 (1.17)	-0.95 (1.18)	0.09 (1.14)	-0.05 (1.13)
Z-score Lumbar spine	-0.88 (1.16)	-0.29 (1.12)	-0.09 (1.05)	0.31 (1.05)
Trabecular bone score	1.34 (0.13)	1.37 (0.06)	1.44 (0.08)	1.42 (0.07)
T-score TBS	-1.00 (1.18)	-1.10 (0.69)	-0.14 (0.77)	-0.56 (0.74)
Cortical vBMD (mg/cm3)	147.50 (21.98)	140.71 (29.85)	172.90 (22.51)	156.46 (20.32)
Cortical vBMD T-score	-1.08 (0.92)	-0.86 (1.41)	-0.01 (0.94)	-0.05 (1.05)
Cortical vBMD Z-score	-1.01 (0.89)	-0.84 (1.40)	0.02 (0.96)	-0.01 (1.06)
Trabecular vBMD (mg/cm3)	172.98 (45.11)	171.64 (53.79)	202.27 (52.36)	187.25 (38.09)
Trabecular vBMD T-score	-1.20 (1.14)	-0.91 (1.43)	-0.46 (1.38)	-0.51 (0.99)
Trabecular vBMD Z-score	-0.56 (0.95)	-0.35 (1.24)	0.14 (1.18)	-0.03 (1.02)
[1] Mean (SD)				

Abbreviations: BMD: bone mineral density; TBS: Trabecular bone score; vBMD: volumetric bone mineral density;
